# Untapped Potential of Unobtrusive Observation for Studying Health Behaviors

**DOI:** 10.2196/46638

**Published:** 2024-02-21

**Authors:** Jack S Benton, David P French

**Affiliations:** 1 Manchester Centre for Health Psychology Division of Psychology and Mental Health The University of Manchester Manchester United Kingdom

**Keywords:** health behavior, environments, context, unobtrusive observation, video technology, computer vision

## Abstract

Improving the environment is an important upstream intervention to promote population health by influencing health behaviors such as physical activity, smoking, and social distancing. Examples of promising environmental interventions include creating high-quality green spaces, building active transport infrastructure, and implementing urban planning regulations. However, there is little robust evidence to inform policy and decision makers about what kinds of environmental interventions are effective and for which populations. In this viewpoint, we make the case that this evidence gap exists partly because health behavior research is dominated by obtrusive methods that focus on studying individual behavior and that are less suitable for understanding environmental influences. In contrast, unobtrusive observation can assess how behavior varies in different environmental contexts. It thereby provides valuable data relating to how environments affect the behavior of populations, which is often useful knowledge for effectively and equitably tackling population health challenges such as obesity and noncommunicable diseases. Yet despite a long history, unobtrusive observation methods are currently underused in health behavior research. We discuss how developing the use of video technology and automated computer vision techniques can offer a scalable solution for assessing health behaviors, facilitating a more thorough investigation of how environments influence health behaviors. We also reflect on the important ethical challenges associated with unobtrusive observation and the use of these emerging video technologies. By increasing the use of unobtrusive observation alongside other methods, we strongly believe this will improve our understanding of the influences of the environment on health behaviors.

## Introduction

It is now widely recognized that features of the environment (eg, green spaces, transport systems, and land use) can shape human behavior [1]. This has led to increasing research and policy interest in the idea that environmental change can be used as an upstream intervention to influence health behaviors, such as physical activity, diet, smoking, and alcohol consumption [2]. Despite the intuitive appeal of this idea, there is a shortage of robust evidence on the effects of environmental interventions [3,4]. A key reason for this is that studies often rely on obtrusive methods for measuring health behavior, which require direct elicitation of information from participants through measures such as self-report (eg, questionnaires), wearable devices (eg, accelerometers), and clinical indicators of behavior (eg, heart rate).

In this viewpoint, we argue that increased use of unobtrusive methods, where measurement does not involve the elicitation of information from participants, is needed to accelerate progress in understanding how environments influence health behaviors. We make the case for unobtrusive observation and discuss the opportunities and ethical challenges associated with the application of video technology and automated computer vision techniques, which could unlock the untapped potential of these underused methods.

## Limitations of Obtrusive Methods for Studying Health Behaviors

Studies on human behavior are dominated by traditional obtrusive methods that focus on understanding individual behavior, often overlooking the broader environmental context. For example, typical studies examining interventions to increase physical activity involve interventions delivered to individuals (eg, in primary care) and rely on obtrusive methods to measure physical activity (eg, self-report, pedometers, and accelerometers) [5]. These studies typically focus on individual behavior irrespective of location, rather than understanding how populations behave in environments, that is, place-based behaviors. However, if we are to effectively and equitably tackle population health challenges such as obesity and noncommunicable diseases, interventions must focus on changing the environmental determinants in populations instead of trying to change each person individually [6].

Research on 3 widely studied health behaviors (alcohol, smoking, and diet) illustrates the importance of focusing on environments. Studies using unobtrusive objective data on sales of alcoholic beverages [7], cigarettes [8], and unhealthy food [9] have tested the effects of policy and environmental interventions, often through evaluating “natural experiments,” that is, real-world interventions where the researcher has no control over the design and delivery of the intervention [10]. Fiscal measures (eg, taxation), policy and legislation (eg, smoke-free policies), and environmental changes (eg, food advertising) are all examples of population-level interventions for changing health behaviors that have been found to work quickly and are cost-effective based on studies using unobtrusive measures [11,12]. These studies have explicitly focused on how changes in environments affect population outcomes (eg, overall sales), rather than examining changes in individuals. However, there remains a skeptical attitude toward these kinds of population-level studies, reflecting the belief that associations on an individual level better reflect “true” causal relationships than those on a population level.

Studies of individuals using traditional obtrusive methods have also methodological biases, which arise because they rely on eliciting information from humans, all of whom have constraints in terms of time, attention, and capabilities. Even before a study has begun, the lengthy and burdensome recruitment process typically stretches from identifying and contacting potentially eligible participants, through eligibility assessment, to obtaining informed consent [13]. This substantial burden on participants reduces response rates and increases attrition, therefore producing sampling bias. Moreover, people from already disadvantaged populations (eg, ethnic minority groups and people with low literacy levels) are more likely to be deterred at each stage, albeit unintentionally [14]. This differential recruitment and attrition threatens the generalizability and equity of research findings.

Even if researchers manage to recruit individuals who are both willing and able to participate in research, obtrusive methods are prone to measurement reactivity. Reactivity effects of research participation include change due to being assessed, having views about the desirability of different possible research requirements, and deliberately or unwittingly trying to satisfy researchers [15]. Self-report, which has long been the dominant method for measuring human behavior, is particularly vulnerable to reactivity because it relies on introspection. Therefore, asking participants to self-report their behavior can lead to response biases from memory recall, cognitive difficulties, and social desirability [16]. Although these various biases are well known, researchers often overlook the extent to which research studies are unusual contexts and that participants may react in unexpected ways to what researchers ask them to do [17]. These biases, resulting from “research participation effects,” have the potential to affect study outcomes in ways that undermine the validity and representativeness of research findings.

## Making the Case for Unobtrusive Observation

Unobtrusive methods have long been recommended to avoid these issues associated with humans taking part in research. In their influential book in 1966, Webb et al [18] argued that researchers rely too heavily on traditional obtrusive measures of data collection. They advocated for greater triangulation using both obtrusive (reactive) and unobtrusive (nonreactive) methods together to provide reassurance that research is robust to the different types of bias associated with each method of measurement. Webb et al [18] described four categories of unobtrusive methods: (1) physical traces, (2) archives, (3) simple observation (observing in natural settings), and (4) contrived observation (observing in controlled settings).

The method that we focus on in this paper is simple observation; specifically, nonparticipative observation of human behavior in the context in which it naturally occurs (hereafter referred to as “unobtrusive observation”). We have been involved in over 1000 hours of unobtrusive observation in natural experimental studies of built environment interventions (eg, a new sustainable park) on physical activity and other behaviors (eg, social interactions) [19-21]. Hence, our experiences derive from a positivist approach, producing quantitative data through systematic observation—a structured method of observation using a predefined coding system.

Unobtrusive observation has historically played a crucial role in various fields of study. For example, sociologists such as Whyte [22] and Jacobs [23] have used observation methods to investigate urban life and better understand how people socially interact in public spaces. Similarly, within urban design, unobtrusive observation has been a valuable tool to provide insights for designers to improve the quality of urban landscapes. This is exemplified in Gehl and Svarre’s [24] pioneering work where they observed public spaces and human behavior, and in studies on “desire paths,” which explore informal routes created by individuals seeking shortcuts rather than adhering to designated paths [25]. Ethnographic research on cultures, communities, and social practices also relies heavily on observation methods, often involving researchers immersing themselves in the communities they observe. Additionally, in ethology (the study of animal behavior), prominent researchers such as Lorenz [26] and Chivers and Goodall [27] have conducted extensive field observations to uncover insights into animal behavior and social structures. In health research, unobtrusive observation has been used to assess a range of health behaviors, including physical activity [28], smoking [29], suicidal behaviors [30], handwashing in clinical settings [31], and social distancing [32]. A common theme across all these observation approaches is that behavior is intricately tied to the environment, and a comprehensive understanding of behavior requires consideration of this contextual influence.

A unique strength of unobtrusive observation, in comparison to other unobtrusive methods, is its ability for fine-grained analysis of variations in health behaviors directly within the environments, or places, in which they occur. This provides us with strong insights into how people’s behavior is influenced by the microenvironments to which they are exposed. As a result, unobtrusive observation is particularly useful for evaluating the effectiveness of environmental interventions aimed at changing health behaviors. For example, Petticrew et al [29] used unobtrusive observation to evaluate the before-and-after impact of a Scottish legislative ban on smoking in public places, which allowed for the assessment of smoking behavior in great detail (eg, quantifying characteristics and behaviors of the smokers or nonsmokers, signage, and positioning of smoking materials) and in different environmental settings (eg, bars, bookmakers, and restaurants). In contrast, other unobtrusive measures, such as archival data, typically involve aggregated measures that make it more difficult for researchers to understand how people are exposed to specific environments of interest.

Observations can be conducted in many public settings, such as green spaces, public highways, shops, and bars, therefore providing real-world contexts for studying health behaviors and the impact of interventions designed to change them. Such studies can provide answers to important questions for policy and decision makers, for example: What kinds of green spaces best encourage physical activity? How can healthier food choices be promoted by changing physical microenvironments (eg, by altering the availability of unhealthy foods)? And how can smoke-free policies in public spaces influence smoking behaviors and secondhand smoke exposure? There is currently a small evidence base for these types of environmental interventions, suggestive of potentially large effects on health behaviors, but with considerable uncertainty and limited understanding of processes by which these outcomes are brought about.

Furthermore, as unobtrusive measures do not require explicit recruitment of participants, observations allow studies in a wide range of populations and settings. Therefore, unlike most traditional research that often fails to recruit participants from underserved groups (typically referred to as “hard-to-reach” groups) [33], using unobtrusive observation can produce valuable evidence in underserved populations where evidence is lacking but the need to improve health is the greatest. This is particularly important given that intervention effectiveness may differ between socioeconomically advantaged and disadvantaged populations [34].

## Underused Method in Health Behavior Research

Despite these advantages, unobtrusive observation remains an underused method for studying health behaviors, even though it has been advocated for over half a century [18]. For example, in a recent systematic review of 116 studies that had an explicit focus on how public spaces influence physical activity, leisure activity, and social activity [35], one would expect that unobtrusive observation would be most appropriate because the focus is on the link between the environment (ie, public spaces) and behavior, rather than the person. Despite this, 95 (82%) of the studies included in this review used obtrusive methods, compared to 53 (46%) studies that used unobtrusive behavior observation. More importantly, of the 95 studies that used obtrusive methods, 57 (60%) studies relied on a single outcome measure to assess behavior, mostly relying on a questionnaire. This is particularly problematic because previous research suggests that relying on methods where participants complete measures in nonbehavioral contexts (eg, at home and in laboratories) may underestimate the importance of contextual factors [36]. Therefore, although this systematic review did not compare differences in findings between obtrusive and unobtrusive methods, relying on questionnaires may lead to inaccurate inferences about the relationship between these behaviors and environmental contexts. This example highlights the importance of triangulation between different methods to reduce the risk of threats to validity based on single-measure research. For example, unobtrusive observation is stronger at quantifying place-based behaviors to examine variation between different contexts (where the place is the subject of analysis), while obtrusive measures are necessary for individual-level longitudinal analysis and assessing intrinsic factors (where the person is the subject of analysis).

So why do researchers typically rely on obtrusive methods and overlook unobtrusive observation? An important factor is that unobtrusive observation does not conform to the traditional mainstays of ethical research that prioritize participants’ right to be informed and freely choose to participate in research. A decline in the number of observational studies reported in journals in several fields has been attributed, in part, to the impact of ethical regulation (eg, [37]). Researchers should, of course, always consider the ethical issues involved in the use of unobtrusive measures, balancing wider societal benefits derived from the research against possible harm to participants. Some specific guidelines have been developed to advise on the unique ethical issues raised by the use of unobtrusive observation (eg, [38,39]). These guidelines typically advise that observational research in public settings where those observed would expect to be observed by strangers, and from which no harm could be reasonably supposed to come, does generally not require consent. Nonetheless, researchers must engage with communities to understand any concerns specific to the sociocultural context they are studying and develop contextually specific solutions to minimize the risk of negative responses when conducting unobtrusive observation. For a more detailed discussion of these ethical challenges, see Clark [40].

Another barrier to the use of unobtrusive observation is the need to deploy in-person observers across multiple study sites, which involves substantial staffing, training, observation time, and data entry—all of which limit scalability. It is therefore perhaps unsurprising that researchers often choose the more convenient and familiar option of traditional obtrusive methods, such as questionnaires and surveys, which have become even more accessible with the rapid rise of digital methods.

## Opportunities in Using Video Technology

With advancements in video technology, observation methods are beginning to use video recordings, which could help address the scalability issues associated with in-person observations and thereby increase the uptake of observational methods. Specifically, cameras can be used to collect video recordings in public spaces, which can then be watched and assessed (“coded”) by a researcher. Using cameras removes the need to recruit, train, deploy, and supervise in-person observers. Therefore, camera-based observations can overcome issues of observer availability, fatigue, and inattention; reduce risks to researchers from working alone and at night for prolonged periods during observations; reduce measurement reactivity associated with the physical presence of observers (JS Benton et al, unpublished data, 2024); and ultimately decrease costs. Furthermore, the ability to pause, rewind, and rewatch footage can improve the reliability of coding (JS Benton et al, unpublished data, 2024) and allow for more in-depth analysis compared with “live” in-person observations.

Although rare, there are examples of camera-based observation research, such as the use of closed-circuit television (CCTV) surveillance to assess bystander behavior in public spaces [41], traffic webcams to assess physical activity [42], drones to assess park use [43], and wearable video devices to assess behavior on sidewalks or streets [44]. The level of unobtrusiveness associated with these various camera-based approaches will depend on the research context. For example, a recent study found no evidence of participant reactivity to the deployment of fixed video cameras in public spaces where there was already existing CCTV surveillance (JS Benton et al, unpublished data, 2024). However, there may be an increased risk of reactivity in public spaces where cameras might be more conspicuous, for example, due to sociocultural norms.

It is difficult to ignore the emergence of new technologies, such as internet of things devices, artificial intelligence, laser tracking, and remote electroencephalography, which are opening up new avenues of unobtrusive measurement of human behaviors [45,46]. For example, researchers are beginning to capitalize on advances in computer vision to use deep learning models (a subset of machine learning) to automatically detect and recognize behaviors within video images. Examples of diverse applications for automated human behavior recognition include analysis of pedestrian behavior and crowds (eg, monitoring social distancing) [47], detecting when a person falls in a health care facility [48], and evaluating sports performance [49]. Developing such models for assessing health behaviors could dramatically reduce the labor, time, and cost needed to collect data at scale, over extended periods, and with increased consistency across video images compared with human observers.

## Ethical Challenges in Using Video Technology

Capitalizing on these emerging video technologies creates new risks associated with recording images of people in public spaces, rather than just observing them. Privacy, consent, and confidentiality are all important challenges, which are entwined within data protection laws that researchers must comply with when processing video recordings of people in public spaces. Using computer vision models could address issues of privacy by eliminating the need for humans to watch video recordings once the models are developed and validated. However, less is known about the broader ethical and societal implications of this approach. Therefore, further work is required to establish responsible research practices for the use (and nonuse) of these techniques.

We recently attempted to provide recommendations on how camera-based research can be conducted ethically and in line with data protection requirements [50], drawing on our experiences in the United Kingdom of conducting 3 studies using fixed video cameras to assess observable health behaviors in public spaces. Examples of good practice include engaging with local communities to codevelop privacy and cybersecurity solutions to minimize the risk of negative responses; displaying privacy notice signs and participant information sheets to increase transparency and ensure compliance with data protection legislation; having clear reporting procedures in place for any observed illegal activities; and implementing robust cybersecurity measures to prevent personal data from being intentionally or unintentionally compromised (eg, using secure data storage solutions).

However, views on what makes this type of camera-based research ethical or not can change depending on the researcher’s positionality, context, and experience. For example, visual researchers in the United Kingdom are increasingly concerned about heightened ethical scrutiny and regulation [51], whereas in the United States, exemptions under the Code of Federal Regulations 46 allow for certain research activities to bypass extensive ethical oversight. It is therefore important to acknowledge differences in ethical standards across different jurisdictions and physical and sociocultural contexts, which will inevitably evolve over time in response to societal, technological, and cultural changes.

There are also important wider societal debates about the use of cameras in research, particularly concerning CCTV use, given its ubiquity in many urban spaces around the world. While the use of CCTV in research is on the rise [52], there are differences between using CCTV footage as an observational method in research and its broader application for public safety. A recent study explored the acceptability of using CCTV for research on suicide prevention, which found that there were positive public attitudes toward this approach [53]. Further research is needed to examine acceptability in different geographical and sociocultural settings and in other areas of health research.

## Conclusions

Understanding how environments influence health behaviors requires a major change in research practices to address our overreliance on obtrusive methods that primarily focus on understanding individual behavior and that tend to overlook environmental influences. Unobtrusive observation can assess how environments affect the behavior of populations; yet despite a long history, it remains an underused method in health behavior research. Capitalizing on video technology and automated computer vision techniques could provide a scalable solution to increase the uptake of these methods. However, we must find a way to ensure that the scientific and societal benefits are maximized while protecting individual rights. By increasing the use of unobtrusive observation alongside other methods, we strongly believe that this will improve our understanding of the influences of the environment on health behaviors.

## Abbreviations

CCTV: closed-circuit television
